# Uncovering the Genetic of Cadmium Accumulation in the Rice 3K Panel

**DOI:** 10.3390/plants11212813

**Published:** 2022-10-22

**Authors:** Chien-Hui Syu, Ting-Iun Nieh, Meng-Ting Hsieh, Yu-Ching Lo, Pei-Rong Du, Yu-Wen Lin, Dong-Hong Wu

**Affiliations:** 1 Agricultural Chemistry Division, Taiwan Agricultural Research Institute, Council of Agriculture, Executive Yuan, Taichung City 413008, Taiwan; 2 Crop Science Division, Taiwan Agricultural Research Institute, Council of Agriculture, Executive Yuan, Taichung City 413008, Taiwan

**Keywords:** *Oryza sativa* L., cadmium accumulation, 3K Rice Genome Project (3K-RGP), allele mining, SNP-Seek

## Abstract

Because Cadmium (Cd) is harmful to humans, and most non-smokers are exposed to Cd mainly through rice consumption, low-Cd rice breeding is urgently needed. It might not be possible to apply variation created using gene editing technology to breeding directly, so it is important to explore genetic variation in this trait in a natural population. In this study, variation in 4 genes was identified among 3024 accessions from the International Rice Research Institute 3000 Rice Genome Project (IRRI 3K-RGP) and 71 other important varieties, and the relationships between the variants and plant Cd accumulation were validated with hydroponic and pot experiments. Variants in *OsNRAMP1*, *OsNRAMP5*, *OsLCD*, and *OsHMA3* were grouped into two, four, three, and two haplotypes, respectively. Fourteen combinations of these haplotypes, which were referred to as Cd-mobile types, were found in the collection. Of these, type 14 was shown to have the greatest potential for low-Cd accumulation, and functional markers for this type were designed. The results of this study provide an important resource for low-Cd rice breeding and highlight an effective strategy for pre-breeding programs.

## 1. Introduction

Cadmium (Cd) is a heavy metal that is harmful to many human organs, such as the kidney and liver, and it can affect the immune system and fertility and even cause cancer [1,2,3,4]. Most non-smokers are mainly exposed to Cd through ingestion. Rice (*Oryza sativa* L.), an important staple food with an average Cd content of about 0.025 mg kg^−1^, is one of the main sources of Cd intake [5,6]. Rice plants take up Cd primarily from soils, and Cd contamination of soil has increased with industrial development [6]. Therefore, to ensure food safety, there is an urgent need to develop approaches to reduce Cd uptake such as developing low-Cd rice varieties [7].

In addition to reducing the bioavailability of Cd in soil through agricultural practices such as water management and soil pH adjustment [8], low-Cd rice breeding is an important strategy [9]. There are three main processes responsible for the accumulation of Cd in rice grains. First, Cd is absorbed by roots. Second, Cd is loaded into and transported through the xylem. Finally, Cd is transferred from the xylem to phloem at nodes and undergoes internode phloem transport [10,11,12]. Although the molecular mechanisms underlying these processes have not been revealed, several genes related to Cd accumulation have been identified. *OsNRAMP1* and *OsNRAMP5*, genes in the natural resistance-associated macrophage protein (NRAMP) family, were shown to play important roles in Cd uptake [7,13]. Both encode transporters located on the plasma membrane and play a role in Cd uptake and transport; unlike OsNRAMP1, which is located on the plasma membrane, *OsNRAMP5* is localized at the distal side of the exodermis and endodermis cells in roots [14,15]. *OsHMA3*, which belongs to another gene family, Heavy Metal ATPase (HMA), is involved in Cd translocation, and encodes a transporter located at the tonoplast in root cells [16]. Miyadate et al. [17] suggested that Cd is sequestered into vacuoles in root cells through an *OsHMA3*-mediated pathway, resulting in restricted Cd transport from roots to shoots. *OsHMA2*, a homolog of *OsHMA3*, is also believed to be involved in root-to-shoot Cd transport [18]. It is localized to the plasma membrane of pericyclic cells in roots and the phloem parenchyma and companion cells in nodes, and it mediates the loading of Cd into xylem and phloem [19]. Low-affinity Cation Transporter 1 (*OsLCT1*) and Low Cadmium (*OsLCD*) were shown to be important for grain Cd accumulation. *OsLCT1* encodes a plasma membrane protein and an efflux-type transporter mainly expressed in leaf blades and nodes [20]. *OsLCD* encodes a protein expressed in vascular tissue, and it was suggested to participate in Cd transport [21].

In addition to validating the functions of these genes by performing experiments on transgenic lines or gene-edited rice, researchers have mapped several quantitative trait loci (QTLs) for Cd-accumulation in grains in segregating populations. For example, *qCd1*, *2*, *3*, *7*, *9*, and *10* were mapped in an F_6_ recombinant inbred line (RIL) population derived from a cross between Bala (*indica*) and Azucena (*japonica*) [22]; *qGCd2* and *qGCd7* were mapped in a backcross BC_1_F_2_ population derived from the recurrent parent Sasanishiki (*japonica*) and donor parent Habataki (*indica*) [23]; *qCd7* was mapped in an F_2_ population derived from Cho-Ko-Koku (*indica*) and Akita 63 (*japonica*) [24]; *qCdp7* was mapped in a BC_1_F_6_ population derived from the recurrent parent Koshihikari (*japonica*) and donor parent Jarjan (*indica*) [25]; and an unnamed QTL was mapped in an F_2_ population derived from Anjana Dhan (*indica*) and Nipponbare (*japonica*) [26]. Interestingly, many of these QTLs mapped to the short arm of chromosome 7, and *OsNRAMP1*, *OsNRAMP5*, and *OsHMA3* are also located in this region [7,16,26]. Therefore, this region is regarded as a hot spot of Cd-accumulation genes. These findings revealed the rich genetic diversity in Cd accumulation between varieties, which would benefit low-Cd accumulation rice breeding. 

Several studies have been dedicated to producing low-Cd rice based on these findings, but few studies have screened the genetic diversity of Cd accumulation-related genes in natural populations. Tian et al. [27] constructed three *LHZ* transgenic rice lines co-expressing *OsLCT1*, *OsHMA2*, and *OsZIP3* and found that Cd accumulation was lower in the *LHZ* lines compared with that in wild type. In another study, *OsNRAMP5* was knocked out using clustered regularly interspaced short palindromic repeat/Cas9 (CRISPR/Cas9) technology, and the Cd concentrations in shoots, roots, and grains of the resulting transgenic *indica* rice lines were lower than those in wild type [28]. Yang et al. [29] also used CRISPR/Cas9 technology to knock out *OsNRAMP5*, and in the resulting transgenic *japonica* rice lines, Cd accumulation was reduced in flag leaves and grains. However, other agronomy traits were also affected, which slightly reduced the grain yield of these plants. To solve this problem, the authors suggested that soil pH and soil water be monitored carefully when planting the *OsNRAMP5* knockout rice to maintain the manganese (Mn) availability in the soil; this is because *OsNRAMP5* was also found to be the major transporter participating in transport of this essential micronutrient. Wang et al. [30] constructed three sets of near isogenic lines (NILs) with marker-assisted backcrossing (MABC) for which the donor was the *japonica* variety IRTA129 and the recipients were the *indica* varieties 9311, H611, and H819. They determined the *OsHMA3-OsNRAMP5-OsNRAMP1* haplotype of each plant, and eight plants from a single NIL were chosen. These plants were homozygous for the IRTA129-type *OsHMA3-OsNRAMP5-OsNRAMP1* haplotype and showed lower Cd and higher Mn contents in brown grains than NILs with other haplotypes. They also showed improvement in some agronomic traits, including days to heading, number of panicles per plant, and yield per plant.

The development of sequencing technology has enabled the generation of a large amount of rice genetic data, which is now shared on public databases, such as the National Center for Biotechnology Information (NCBI, https://www.ncbi.nlm.nih.gov/, accessed on 1 January 2020), Rice Annotation Project Database (RAP-DB, https://rapdb.dna.affrc.go.jp/, accessed on 1 January 2020), and Rice SNP-Seek Database (https://snp-seek.irri.org/, accessed on 1 January 2020). This allows scientists to easily share genetic resources and greatly benefits plant research.

In our previous study, we constructed a low-Cd *indica* line, TCS10-*OsNRAMP1*, using MABC. Specifically, the low Cd uptake allele of *OsNRAMP1* was introgressed from the *japonica* cultivar Tai Keng 2 (TK2) into the *indica* cultivar Taichung Sen 10 (TCS10). The level of Cd accumulation in TCS10-*OsNRAMP1* was 42.2% lower than that in TCS10, but other traits observed in TCS10-*OsNRAMP1* were similar to those in TCS10; this line reduces the risk of Cd intake from *indica* rice consumption and promotes food safety [31]. To identify more genetic resources for low-Cd rice breeding, here we identified variation in four genes, *OsNRAMP1*, *OsNRAMP5*, *OsLCD*, and *OsHMA3*, in 71 varieties that are popular or important in Taiwan and 3024 rice accessions from the International Rice Research Institute 3000 Rice Genome Project (IRRI 3K-RGP, https://snp-seek.irri.org/_snp.zul, accessed on 1 January 2020). Then, the association between haplotype and plant Cd uptake and accumulation was investigated, and functional markers were designed.

## 2. Results

### 2.1. Genotype Organization

**Figure 1:**
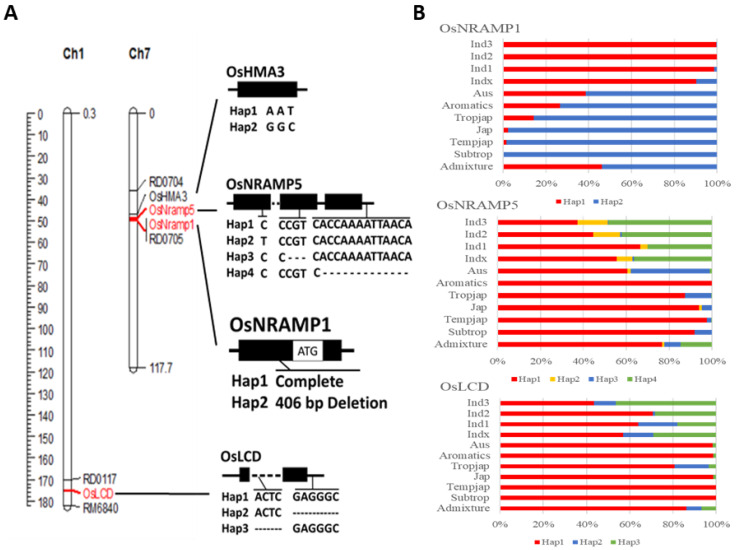
The haplotypes of *OsHMA3*, *OsNRAMP1*, *OsNRAMP5*, and *OsLCD* (**A**) and their distributions among 3K-RGP accessions (**B**). Subpopulation designation is based on Alexandrov et al. (2015) [33]. Ind1, ind2 and ind3 are three groups of *indica* rice, indx corresponds to other *indica* varieties, temp is temperate *japonica*, trop is tropical *japonica*, temp/trop and trop/temp are admixed temperate and tropical *japonica* varieties, japx is other *japonica* varieties, Aus is *aus*, inax is admixed *aus* and *indica*, Aromatics is aromatic and admixture is all other unassigned varieties.

Four genes were used as queries in searches of the IRRI 3K-RGP dataset, where Nipponbare was defined as the reference genome. *OsHMA3* (Os07g0232900), *OsNRAMP1* (Os07g0258400), and *OsNRAMP5* (Os07g0257200) are located in the major QTL *qCdT7* on chromosome 7, and *OsLCD* (Os01g0956700) is located on chromosome 1. Using the sequence data for 3024 varieties, genotypes of these genes were grouped into several haplotypes (Figure 1A). Genotypes with complete sequences, which were related to high Cd accumulation, were defined as Hap1; all other haplotypes were responsible for low Cd accumulation phenotypes. *OsHMA3* was found to be 2082 bp long, consist of 13 exons and 12 introns, and have 5′- and 3′-untranslated regions (UTRs) with lengths of 84 bp and 441 bp, respectively. The *OsHMA3* genotypes could be divided into two haplotypes, ATT and GGC, according to a SNP in exon 1. However, none of the easily transportable genotypes described in Yan et al. (i.e., any combination of the following three variants: substitution at amino acid 80, substitution at amino acid 380, and insertion–deletion (InDel) at amino acids 826–878) [32] were found.

*OsNRAMP1* was found to be 2995 bp long, consist of 13 exons and 12 introns, and have 5′- and 3′-UTRs with lengths of 266 bp and 376 bp, respectively. Genotypes of *OsNRAMP1* were divided into two haplotypes according to a 406 bp deletion in the promoter. There was a clear difference in the distributions of the two haplotypes between the two major subspecies *indica* and *japonica*. The frequency of *OsNRAMP1*-Hap1 in *indica* was higher than 90%, while that of *OsNRAMP1*-Hap2 in *japonica* was higher than 85% (Figure 1B, Table S2).

*OsNRAMP5* was found to be 2006 bp long, consist of 13 exons and 12 introns, and have 5′- and 3′-UTRs with lengths of 96 bp and 293 bp, respectively. Genotypes of *OsNRAMP5* were classified into four haplotypes. *OsNRAMP5*-Hap2 had a C/T SNP in exon 13, *OsNRAMP5*-Hap3 had a 3 bp deletion in exon 7, and *OsNRAMP5*-Hap4 had a 13 bp deletion in intron 13. *OsNRAMP5*-Hap1 and *OsNRAMP5*-Hap4 were the most common haplotypes, with frequencies of 65.58% and 23.64%, respectively, while *OsNRAMP5*-Hap2 and *OsNRAMP5*-Hap3 were mainly found in *indica* and Aus, with overall frequencies of 5.56% and 5.16%, respectively (Figure 1B, Table S2). *OsLCD* was found to be 1775 bp long and consist of eight exons. The 5′-UTR consists of three exons 191 bp, 170 bp, and 83 bp in length, and the 3′-UTR consists of two exons 23 bp and 355 bp in length. Genotypes of *OsLCD* could be divided into three haplotypes. *OsLCD*-Hap2 had a 6 bp deletion near the 5′-UTR, and *OsLCD*-Hap3 had a 4 bp deletion near the 3′-UTR. The frequencies of OsLCD-Hap1 in the *indica* and *japonica* varieties were 40% and 70%, respectively, and the remaining 60% of indica varieties shared *OsLCD*-Hap2 and *OsLCD*-Hap3 (Figure 1B, Table S2).

In addition to the 3K-RGP accessions, 71 important varieties were also analyzed for genotypes of *OsNRAMP1*, *OsNRAMP5*, and *OsLCD*. The distribution of haplotypes in these 71 varieties was similar to that in 3K-RGP accessions. The frequency of *OsNRAMP1*-Hap1 in *indica* was 94% and that of *OsNRAMP1*-Hap2 in *japonica* was 81%. The *OsNRAMP5* haplotypes found in most of the varieties were *OsNRAMP5*-Hap1 and *OsNRAMP5*-Hap4, and their distributions in *indica* and *japonica* varieties were similar. The frequencies of *OsNRAMP5*-Hap1 and *OsNRAMP5*-Hap4 were 77% and 16%, respectively; those of *OsLCD*-Hap1 in *indica* and *japonica* varieties were 38% and 94%, respectively; and the remaining 62% of *indica* varieties contained *OsLCD*-Hap2 and *OsLCD*-Hap3 (Table S2).

According to the *OsNRAMP1*, *OsNRAMP5*, and *OsLCD* genotypes, the 3K-RGP germplasm were grouped into 14 types, were referred as Cd-mobile types, related to different abilities to transport and accumulate Cd. *OsNRAMP1*-Hap1 was found in *OsNRAMP1* type1 to type7, and *OsNRAMP1*-Hap2 was found in types 8 to 14. Among the 14 types, types 12 to 14 were low Cd accumulation types, and did not possess the Hap1 allele for any of the three genes. These 14 types covered 92% of the 3K-RGP germplasm and were considered representative. Type1, type5, and type8 were the three major types, with frequencies of 20%, 14%, and 27%, respectively. Most of the *indica* and *japonica* accessions belonged to these types. The frequencies of type1 and type5 *indica* subspecies were both 20%, and the frequency of type8 *iaponica* subspecies was 90%. The remaining 60% of *indica* accessions belonged to the other types, mainly type3 and type7, and the remaining *japonica* accessions mainly belonged to type11. The subspecies *Aus* mainly consisted of type1, type8, and type11 accessions, while Aromatics mainly consisted of type1 and type8 accessions. Other minor Cd-mobile types, such as type2, type6, and type9, were scattered among different subspecies (Table S3).

### 2.2. Correlation Analysis of Cd-Mobile Types and Cadmium Accumulation

**Figure 2:**
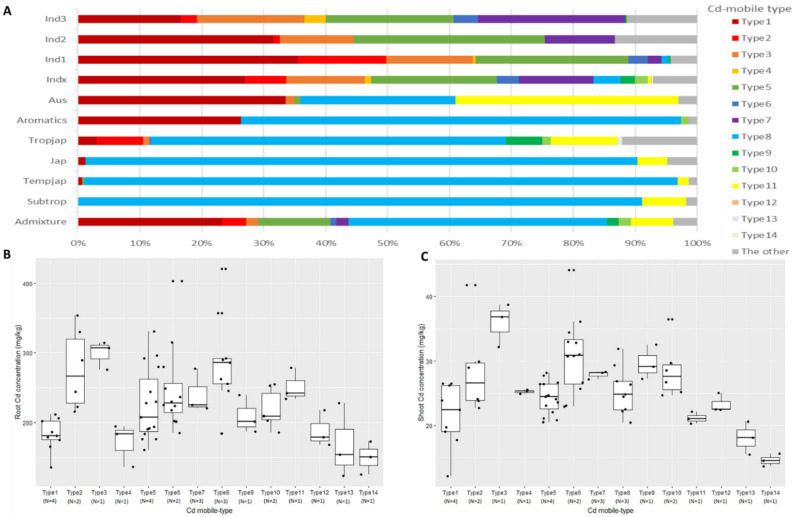
The distribution of 14 Cd-mobile types among the 3K-RGP accessions (**A**), and the Cd concentrations in roots (**B**) and shoots (**C**) in a hydroponic experiment. Ind1, ind2 and ind3 are three groups of *indica* rice, indx corresponds to other *indica* varieties, temp is temperate *japonica*, trop is tropical *japonica*, temp/trop and trop/temp are admixed temperate and tropical *japonica* varieties, japx is other *japonica* varieties, Aus is *aus*, inax is admixed *aus* and *indica*, Aromatics is aromatic and admixture is all other unassigned varieties. N, the number of varieties. Each variety has three replicates and labels the original points in the graph.

To understand the Cd absorption and transport capacity of the 14 Cd-mobile types, a hydroponic experiment was conducted, and the results are shown in Table 1. The concentrations of Cd in roots of type2, type3, and type6 accessions were higher than 270 mg kg^−1^, and the concentrations in type3 (299 mg kg^−1^) accessions was the highest among the 14 types. However, for type12, type13, and type14, Cd concentrations in roots were less than 200 mg kg^−1^, and those in type14 were the lowest, only half of those in type3. The shoot Cd concentrations in type3 and type6 accessions reached 35 mg kg^−1^, while those in type13 and type14 accessions were lower. We also observed that Cd concentrations in shoots of type14 were approximately half as much as those in type3. The highest and the lowest shoot-Cd/root-Cd ratios were observed in type4 and type11 accessions, which had ratios of 14.75% and 8.41%, respectively (Table 1, Figure 2).

All type1 to type11 accessions had the Hap1 genotype, which is related to the high Cd accumulation phenotype, for at least one of the three genes. The Cd concentrations were higher than 200 mg kg^−1^ in roots and 20 mg kg^−1^ in shoots. In contrast, the Cd concentrations in type12, type13, and type14 accessions were lower than 200 mg kg^−1^ in roots and 20 mg kg^−1^ in shoots. The difference between the two groups was significant, confirming that type12, type13, and type14 accessions have low Cd accumulation potential, with type14 having the lowest (Table 1).

To verify the capacity of type14 accessions to absorb and accumulate Cd, a pot experiment was conducted with soil collected from a Cd-contaminated paddy field. The Cd concentration of this soil was 3.5 mg kg^−1^ (Table S5), which is between 2.5 mg kg^−1^ and 5 mg kg^−1^, the Taiwan Environmental Protection Administration monitoring and control standard for farmland soils. Of the five representative varieties used in the pot experiment, TN11 and TCS10-OsNRAMP1 belonged to type8, and Uprh166, Landeo, and Asu belonged to type11, type14 and type13, respectively. Type11, type13 and type14 were combinations of three different haplotypes of *OsLCD*, *OsNRAMP1*-Hap2, and *OsNRAMP5*-Hap3 (Table 2).

The Cd concentrations in brown rice of all varieties grown in the pot experiment were far lower than 0.4 mg kg^−1^, the safety value for edible rice defined by the Ministry of Health and Welfare of Taiwan. The concentration was the lowest for Asu (0.03 mg kg^−1^) and the highest for Uprh166 (0.16 mg kg^−1^). Similar trends were observed for root and shoot Cd concentrations, which were 4.98 mg kg^−1^ and 0.68 mg kg^−1^, respectively, for Asu, and 22.54 mg kg^−1^ and 1.33 mg kg^−1^, respectively, for Uprh166. There was no significant difference in the concentrations between different parts of the plants. This result confirmed that type14 is a Cd-mobile type with low Cd accumulation potential, which is consistent with the results of the hydroponic experiment (Figure 2, Table 2).

### 2.3. Specific Marker Design and Genotype Analysis

**Figure 3:**
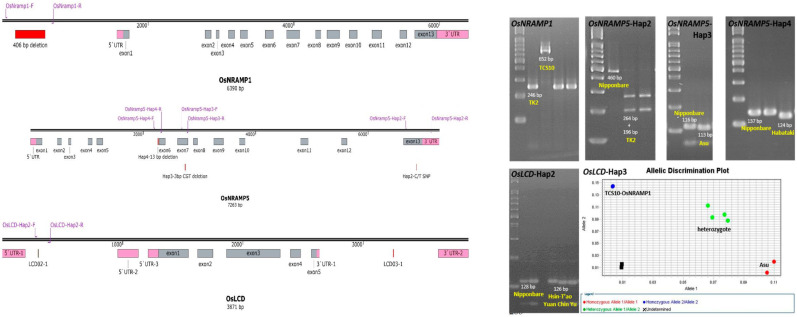
The gene structures, functional markers, and PCR results for haplotypes of *OsNRAMP1*, *OsNRAMP5*, and *OsLCD*.

To improve the utilization of natural resources for modulation of Cd absorption and transport, functional markers were designed for *OsNRAMP1*, *OsNRAMP5*, and *OsLCD*. An InDel marker was designed for the 406 bp deletion in the promoter of *OsNRAMP1*. For *OsNRAMP5*, three markers, *OsNRAMP5*-Hap2, *OsNRAMP5*-Hap3, and *OsNRAMP5*-Hap4, were designed to detect different haplotypes. The *OsNRAMP5*-Hap2 reaction was designed to detect the C/T SNP; the amplified products of *OsNRAMP5*-Hap2 were digested by HpyCh4III to produce 264 bp and 196 bp fragments, while the products of other haplotypes were 460 bp long. The Tm for the *OsNRAMP5*-Hap2 reaction was 60 ℃. There were 3 bp and 13 bp deletions in *OsNRAMP5*-Hap3 and *OsNRAMP5*-Hap4, respectively. In the *OsNRAMP5*-Hap3 reaction, the amplified products were 113 bp long, and those of the other haplotypes were 116 bp long. In the *OsNRAMP5*-Hap4 reaction, the amplified products were 124 bp long, and those of the other haplotypes were 137 bp long. The Tm values of the *OsNRAMP5*-Hap3 and *OsNRAMP5*-Hap4 reactions were both 55 ℃. For *OsLCD*, two markers, *OsLCD*-Hap2 and *OsLCD*-Hap3, were designed to detect the 6 bp and the 4 bp deletions in *OsLCD*-Hap2 and *OsLCD*-Hap3, respectively. In the *OsLCD*-Hap2 reaction, the amplified products were 122 bp long, while those of the other haplotypes were 128 bp long. A TaqMan marker was used to distinguish *OsLCD*-Hap3 from the other haplotypes by detecting the 4 bp deletion (Figure 3, Table S4).

## 3. Discussion

The major QTL qCdT 7, which is 1.6 Mb long on chromosome 7, contains three genes related to Cd accumulation in rice plants, namely *OsNRAMP1*, which is responsible for Cd uptake by roots, *OsHMA3*, which is responsible for Cd transport, and *OsNRAMP5*, which is responsible for Cd, iron (Fe), and Mn uptake by roots [13,16,17,21,26,34,35]. These genes and another Cd accumulation-related gene, *OsLCD*, were chosen as target genes, and variants of these genes were summarized in this study. The 14 Cd-mobile types discussed here were organized according to the haplotypes of *OsNRAMP1*, *OsNRAMP5*, and *OsLCD*. Previous research showed that the deletion in *OsNRAMP1*-Hap2 resulted in the excessive expression of *OsNRAMP1* and an increase in Cd absorption, and that the negative regulation of *OsNRAMP1*-Hap1 inhibited the uptake of Cd by roots [13]. In another study, Yan et al. found that loss of function of *OsHMA3* leads to high Cd accumulation in shoots and grains [32], but here no loss-of-function allele was found. However, the possible influence of *OsHMA3* should be considered carefully because *OsNRAMP5* and *OsHMA3* are located in close proximity and their relationship is still unclear. It is possible that a weak allele of *OsNRAMP5* combined with a strong allele of *OsHMA3* leads to the reduction in Cd accumulation in rice grains [9].

In the past decade, artificial variants have been created using a variety of methods, such as carbon ion-beam irradiation [7], T-DNA insertion [21,36], RNA interference [35,37], and CRISPR/Cas9 [28,38]. However, the application of these variants in low-Cd rice breeding has led to different results. Knockout of *OsNRAMP5* reduces Cd accumulation and plant growth simultaneously [29] but the low-Cd accumulation plants have lower rice yield [28]. This indicates that artificial mutations can be used to generate ideal plants, but these plants might have unknown disadvantages. In contrast, Sun et al. [39] found that natural variants related to grain Cd content were not associated with the contents of other heavy metals such as Fe, Mn, copper (Cu) and Zinc (Zn), suggesting that breeding low-Cd rice using natural variation would not disrupt absorption of other elements or plant growth. Therefore, it is important to assess the genetic diversity of natural populations. Some variants related to Cd transport from shoot to grain were found in a previous study of 49 rice varieties [40]. A large deletion in the *OsNRAMP1* promoter in Habataki was found to lead to a higher Cd content than that in Nipponbare [32]. Mutations in the 80th and 380th amino acids and a 153 bp deletion in 7th exon of *OsHMA3* were found to affect functions related to Cd accumulation [16,17,32]. An InDel and 35 SNPs found in *OsNRAMP5* were thought to aid low-Cd rice breeding [41]. Wang et al. [30] also reported that introduction of the *OsHMA3-OsNRAMP5-OsNRAMP1* fragment from IRAT129 into 9311 resulted in a reduction in Cd accumulation in rice grains. In the present study, the 3K-RGP accessions were assigned to types according to genetic diversity in *OsNRAMP1*, *OsNRAMP5*, and *OsLCD*, which provides a valuable resource for low-Cd rice breeding in the future.

Variation of Cd accumulation in a natural population has been explored in other ways in previous studies. Several major QTLs were detected in a single hotspot among different mapping populations [23,26,42,43,44]. For example, both *qCdp7* and *qCdT7* are located on chromosome 7; *qCdp7* explained 31–51% of the variation of Cd content in brown rice and 46–54% of the content in shoots [25], and *qCDT7* explained 88% of variation in Cd transport [24]. Some QTLs were also detected in a genome-wide association study (GWAS) [45]. However, it takes a lot of time to develop a population for QTL mapping, and false positives frequently occur in GWAS, requiring additional verification. Searching for variations in known genes provides a rapid and promising way to breed low-Cd rice. For example, two variants of *OsNRAMP1* and *OsNRAMP5* were found to explain 46.4% and 22.6% of the variation in grain Cd accumulation, respectively, in a screen of seven Cd-related genes among 174 thermosensitive genic male sterile lines [46]. In this study, three Cd-mobile genotypes with the potential for low Cd accumulation were found and could be applied to breeding immediately.

In addition to the four genes discussed in the present study, large numbers of genes have also been shown to be involved in Cd uptake and transport. *OsIRT1* and *OsIRT2* transport Fe together with Cd [47]. *OsHMA2* transports Cd and Zn from roots to shoots [48]. The plasma membrane protein *OsMTP1* is also involved in transport of Zn and Cd. *OsABCG43* confers Cd tolerance in yeast, which indicates that it might also affect Cd accumulation in rice [49]. The transporter *OsLCT1* transports Cd in phloem [20]; however, Songmei et al. [38] showed that the reduction in Cd accumulation caused by a mutation in *OsLCT1* was less than that observed when *OsNRAMP5* was mutated, which suggested that *OsNRAMP5* plays a larger role in Cd accumulation than *OsLCT1*. In addition, *OsNRAMP1*, *OsNRAMP5*, and *OsLCD* explained nearly 79.3% of variation of Cd accumulation observed in our study (Table S5), which indicates that these three genes are responsible for most of the variation in Cd accumulation in rice plants.

The ability of rice plants to take up and accumulate Cd can be verified by performing hydroponic, pot, and field experiments [13,21,29,32,36,39,40,41,46]. In this study, Cd concentrations in roots and shoots were first analyzed in hydroponic experiments among 14 Cd-mobile types, and then verified for 5 representative varieties by performing pot experiments together with measurements of Cd concentration in brown rice. Functional markers based on PCR and agarose gel electrophoresis were designed; these markers are easy to use and offer a rapid, specific, and easy genotyping method for future studies.

## 4. Materials and Methods

### 4.1. Gene Data Collection

The gene name was used to search for gene ID, and the gene ID was converted into the MSU form in RAP-DB. The gene ID was then used to search gene loci and sequences in IRRI 3K-RGP, which consists of 3024 accessions from the International Rice Genome Sequencing Project 1.0 (IRGSP 1.0). The “Autogroup” function on the same website with default parameters was used to identify haplotypes. All results were downloaded with variety names.

### 4.2. Plant Materials

Genotyping of target genes was conducted for 71 varieties, which were either popular cultivars in Taiwan or important germplasm. Three *japonica* rice cultivars, Nipponbare, Tainan 11 (TN11), and Taitung 30 (TT30); four *indica* rice cultivars, Habataki, TCS10, Taichung Sen 17 (TCS17), and Taichung Sen Waxy 2 (TCSW2); and 20 rice accessions introduced from IRRI were used in a hydroponic experiment (Table S1). Five representative varieties were used in a pot experiment, namely three accessions introduced from IRRI, Uprh166, Landeo, and Asu, and two Taiwanese cultivars, TN11 and TCS10-OsNRAMP1. TCS10 and TK2 were used for development of functional markers for *OsNRAMP1*; Habataki, Asu, Nipponbare, and TK2 were used for *OsNRAMP5* marker development; Asu and the *indica* accession Hsin-T’ao Yuan Chin Yu introduced by IRRI were used in marker development for the *OsLCD* locus.

### 4.3. Hydroponic and Pot Experiments

Hydroponic culturing was performed in a greenhouse with a controlled temperature (35/25 °C, day/night) under sunlight. Rice seeds were sterilized in a solution containing 1% sodium hydrochloride and one drop of Tween 20 for 30 min, and then soaked in water for 2 days. Next, the seeds were drained and allowed to rest in an airtight container for 1 day. After germination, 20 seedlings were transferred to an iron mesh set on the surface of culture solution in a 0.6-L beaker. Seedlings were then raised in half-strength modified Kimura B nutrient solution (0.18 mM (NH_4_)_2_SO_4_, 0.09 mM KNO_3_, 0.27 mM MgSO_4_·7H_2_O, 0.09 mM KH_2_PO_4_, 30.6 μM Fe-citrate, 183 μM Ca(NO_3_)_2_·4H_2_0, 2.51 μM H_3_BO_3_, 152 nM MnSO_4_·4H_2_O, 202 nM ZnSO_4_·7H_2_O, 52 nM CuSO_4_·5H_2_O, and 49 nM MoO_3_·H_2_O, pH adjusted to 4.8–5.0, solution renewed every 3 days) for 16 days until they reached the three-leaf stage. Then, the solution was replaced with full-strength nutrient solution and the indicated amount of Cd stock solution was added. The stock solutions of Cd were prepared using a Cd standard solution (1000 g mL^−1^ in 2% HNO_3_, High-Purity Standards). The Cd treatment concentrations were 0 and 0.1 mg L^−1^, and the exposure time was 14 days (growth period: July to August, 2020). Three replicates (pots) for each of the Cd treatments were conducted. After harvesting, the rice seedlings were separated into roots and shoots and rinsed first with tap water and then with deionized water. The biomass and lengths of each root and shoot were measured.

To understand the differences in Cd accumulation in rice grains among varieties with different target gene alleles, representative rice varieties were planted in Cd-contaminated soils. Topsoil (0–20 cm) was collected from paddy soils in Houli District, Taichung City, Taiwan; the paddy soil in this area had suffered Cd contamination caused by irrigation with Cd-containing wastewater in the past. The Cd concentration of the test soil was 3.76 mg kg^−1^. Air-dried soil samples were passed through a 2 mm sieve, homogenized, and stored in plastic vessels. The basic properties of the test soil, namely soil pH [50], organic matter [51], cation exchange capacity [52], the available Cd concentration [53] and the total Cd concentration [54], were analyzed. 

Pot experiments were conducted in the same glasshouse mentioned above. The rice seed sterilization, germination, and seedling-raising procedures were the same as those described for the hydroponic experiment. Three well-grown seedlings were selected and transplanted together into a pot filled with 5 kg of the test soil for ~105 days of cultivation. The water level of the pots was maintained at 3–5 cm above the soil surface to simulate flooding conditions throughout the whole cultivation period. The soils were supplemented with 220 kg of nitrogen (N) ha^−1^, 100 kg of phosphorus oxide (P_2_O_5_) ha^−1^, and 180 kg of potassium oxide (K_2_O) ha^−1^ by the addition of urea (CO(NH_2_)_2_), monocalcium phosphate (Ca(H_2_PO_4_)_2_·H_2_O), and potassium chloride (KCl) as fertilizers. Fifty percent N, 100% P_2_O_5_, and 40% K_2_O were applied as the base fertilizer before transplantation. Twenty-five percent N and 40% K_2_O were applied as the first top dressing 30 days after transplantation, and 25% N and 20% K_2_O were applied as the second top dressing 49 days after transplantation. After harvesting, rice plants were rinsed with tap water and divided with ceramic scissors into roots, shoots, and grains, and then the biomass of plant tissues and grain yield were measured.

### 4.4. Plant Collection, Digestion, and Analysis

Air-dried plant tissues (roots, shoots, brown rice) were digested separately with concentrated HNO_3_ (69–70%, J.T. Baker, Phillipsburg, NJ, USA)/H_2_O_2_ (30% [*w*/*w*] in H_2_O, Sigma-Aldrich, Burlington, MA, USA) in a heating block at 125 ℃ [55]. The digests were diluted by adding deionized water to 50 mL, filtered through a 0.45 μm filter, and stored in plastic bottles for subsequent element analysis. The concentrations of Cd in the digests were determined by inductively coupled plasma-mass spectrometry (ICP-MS 7700, Agilent Technologies, Taipei, Taiwan).

### 4.5. Functional Marker Design and Genotyping

Genomic DNA of leaves was extracted by quick extraction. Leaves (100 mg) were cut into pieces in a 2 mL tube, and 20 μL quick extract buffer (QuickExtract Plant DNA Extraction Solution, QEP70750) was added. The sample was centrifuged for 5 min at room temperature at 4400 rpm and incubated at 65 ℃ for 6 min and 95 ℃ for 2 min on a polymerase chain reaction (PCR) machine (Thermo Fisher Scientific, Waltham, MA, USA). Then, 50 μL of deionized water was added.

Sequences of haplotypes downloaded from RAP-DB were used as queries in a blast search of bacterial artificial chromosome sequences downloaded from NCBI in BioEdit [56], and functional markers were designed using Primer Premier 5 (Premier Biosoft International, Palo Alto, CA, USA) and NCBI Primer Blast [57] (Table S2). Except for *OsLCD*-Hap3, all markers were PCR-based. Each 10 μL PCR reaction contained 1× Taq Buffer, 2 mM MgCl_2_, 0.2 mM dNTP, 0.6 U Taq polymerase (BioVan, Taichung, Taiwan), 0.2 μM forward primer, 0.2 μM reverse primer, and 2 μL sample DNA. The sample was first denatured at 95 ℃ for 1 min, followed by 35 cycles of denaturation for 15 s at 95 ℃, annealing for 15 s at the melting temperature (Tm) (Table S3), and extension for 20 s at 72 ℃, and a final extension for 1 min at 72℃. PCR products were visualized by electrophoresis in a 26 cm × 26 cm 3% Agarose Low EEO gel at 350 V for 30 min. Because the products of *OsNRAMP1*-Hap1 were large, the annealing and extension times were modified to 20 and 25 s, respectively. *OsNRAMP5*-Hap2 was designed as a cleaved amplified polymorphic sequences (CAPS) marker; the PCR products were digested by HpyCh4III overnight at 37 ℃ followed by inactivation at 65 ℃ for 20 min to produce 264 bp and 196 bp fragments. *OsLCD*-Hap3 was designed as a TaqMan marker. The 10 μL reaction sample contained 1× qPCRBIO Probe Blue Mix (PCR Biosystems, London, UK), 1× custom TaqMan SNP genotyping assay (Thermo Fisher Scientific), and 2 μL sample DNA. The sample was incubated at 25 ℃ for 30 s and 95 ℃ for 20 s, followed by 45 cycles of 3 s at 95 ℃ and 20 s at 60 ℃ on a StepOnePlus quantitative PCR machine (Thermo Fisher Scientific), and the data were analyzed with StepOne software v2.3 (Thermo Fisher Scientific).

### 4.6. Statistical Analysis

The outliers were removed before data analysis, and replicates exceeding one standard deviation from the mean were eliminated. Analysis of variance (ANOVA) was performed with the package *agricolae* [58] in R Studio [59]. If differences between varieties and treatments were significant, ANOVA was performed again for single genes or genotypes to divide the degrees of freedom from varieties. Then, the least significant difference test (LSD) was used to determine whether the difference between genotypes was significant, and LSD_0.05_ was calculated. To visualize the results, the package *ggplot2* [60] was used in R Studio.

## 5. Conclusions

In conclusion, haplotypes of *OsHMA3*, *OsNRAMP1*, *OsNRAMP5*, and *OsLCD* were summarized in this study, and their relationships with Cd accumulation in rice plants were verified in hydroponic and pot experiments. Among them, type14 accessions which possess deletions in *OsHMA3*, *OsNRAMP1*, and *OsNRAMP5* were shown to have low Cd accumulation potential, and functional markers for this type were designed. The strategy used in this study provides an effective tool for low-Cd rice pre-breeding.

## Figures and Tables

**Table 1:** The Cd concentrations for representative varieties of 14 Cd-mobile types tested in hydroponic experiments.

Cd Mobile-Type	*NR1*	*NR5*	*LCD*	Cd Concentration (mg kg^−1^)			Variety
				Root ^1^	Shoot	Shoot/Root Ratio	
Type1	Hap1	Hap1	Hap1	195.19 bc	22.64 cd	11.60% abc	Habataki, Taichung Sen 10, Kurulutudu, Maranhao Branco
Type2	Hap1	Hap1	Hap2	275.91 a	28.55 b	10.35% bc	Taichung Sen 17, Chang Le San Shu Zao
Type3	Hap1	Hap1	Hap3	299.13 a	35.91 a	12.01% abc	Hsinchu Ai Chio Chieng
Type4	Hap1	Hap2	Hap2	171.31 de	25.26 cd	14.75% a	Hsi’-T’ao Yuan Ching Yu
Type5	Hap1	Hap4	Hap1	223.47 cde	23.91 ef	10.70% abc	Taichung Sen Waxy 2, Ncs840, Psbrc50, IR 19661-364-1-2-3
Type6	Hap1	Hap4	Hap2	278.80 a	35.19 a	12.62% ab	B 6136-3-TB-0-1-5, B 6136 E-3-TB-0-1-5
Type7	Hap1	Hap4	Hap3	224.41 bc	27.08 bc	12.07% abc	Ncs771A, Arc14868, IR 80310-12-B-1-3-B
Type8	Hap2	Hap1	Hap1	288.23 a	25.42 cd	8.82% c	Nipponbare, Tainan 11, Taitung 30
Type9	Hap2	Hap1	Hap2	209.07 bcde	29.63 b	14.18% ab	Jin Jun Dao
Type10	Hap2	Hap1	Hap3	219.09 bcd	28.54 b	13.03% ab	Lobang (white), Balibud
Type11	Hap2	Hap3	Hap1	251.53 ab	21.16 ef	8.41% c	Uprh166
Type12	Hap2	Hap2	Hap3	188.28 cde	23.32 de	12.39% abc	Ai Jiao Zi
Type13	Hap2	Hap3	Hap2	168.37 de	18.09 fg	10.75% abc	Landeo
Type14	Hap2	Hap3	Hap3	149.88 e	14.64 g	9.77% bc	Asu

^1^ Values are expressed as the mean of three replicates and means within each column followed by the same letter(s) are not significantly different at the 5% level as determined by Fisher’s protected LSD test. *NR1*: *OsNRAMP1*; *NR5*: *OsNRAMP5*; *LCD*: *OsLCD*.

**Table 2:** The Cd concentrations for representative varieties of five Cd-mobile types tested in pot experiments.

Cd in Soil (mg kg^−1^)	Cd-Mobile Type	*NR1*	*NR5*	*LCD*	Cd Concentration (mg kg^−1^)						Cd Concentration Ratio						Variety
					Root ^1^		Shoot		Brown Rice		Shoot/Root		Brown Rice/Root		Brown Rice/Shoot		
3.76	Type8	Hap2	Hap1	Hap1	13.29	b	1.24	ab	0.04	b	9.33%	a	0.30%	b	3.23%	a	TN11
	Type8	Hap2	Hap1	Hap1	7.01	bc	0.68	c	0.07	b	9.70%	a	1.00%	a	10.29%	a	TCS10-OsNRAMP1
	Type11	Hap2	Hap3	Hap1	22.54	a	1.33	a	0.16	a	5.90%	a	0.71%	ab	12.03%	a	Uprh166
	Type13	Hap2	Hap3	Hap2	8.96	bc	0.88	bc	0.06	b	9.82%	a	0.67%	ab	6.82%	a	Landeo
	Type14	Hap2	Hap3	Hap3	4.98	c	0.68	c	0.03	b	13.65%	a	0.60%	ab	4.41%	a	Asu

^1^ Values are expressed as means of three replicates. Letters represent the results of Fisher’s protected LSD test at the 95% confidence level. *NR1*: *OsNRAMP1*; *NR5*: *OsNRAMP5*; *LCD*: *OsLCD*; TN11: Tainan; TCS10-OsNRAMP1: Taichung Sen 10-OsNRAMP1.

## Data Availability

The datasets presented in the study are either included in the article or in the Supplementary Materials; further inquiries can be directed to the corresponding authors.

## Supplementary Materials

The following supporting information can be downloaded at: https://www.mdpi.com/article/10.3390/plants11212813/s1, Table S1: Accession number, subspecies, and nationality of 27 varieties used in hydroponic experiments; Table S2: The frequency (shown as a percentage) of each *OsNRAMP1*, *OsNRAMP5*, and *OsLCD* haplotype in the rice 3K panel; Table S3: The functional markers for each *OsNRAMP1*, *OsNRAMP5*, and *OsLCD* haplotype; Table S4: The frequencies of 14 Cd-mobile types in the 3K-RGP panel; Table S5: Basic properties of the Cd-contaminated paddy soil.
