# Supplementary material for: Uncovering the Genetic of Cadmium Accumulation in the Rice 3K Panel
Source: Plants (Basel). 2022 Oct 22;11(21):2813. doi: 10.3390/plants11212813 (PMC9657585; doi:10.3390/plants11212813)
Supplement: Supplementary file 1 [file plants-11-02813-s001.zip › plants-1908221-supplementary.pdf]

Supplementary

Table S1. Accession number, subpopulation, and nationality of 27 varieties used in hydroponic experiments.

No.	Accession number[1]	Variety	Subpopulation[2]	Nationality
1	IRGC 126251	Ncs840	Ind2	India
2	IRGC 127665	Ncs771A	Ind2	India
3	IRGC 125813	Kurulutudu	Ind2	Sri Lanka
4	IRGC 127963	Arc14868	Ind2	India
5	IRGC 126115	Chang Le San Shu Zao	Ind1	China
6	IRGC 127420	Hsinchu Ai Chio Chieng	Ind1	Taiwan
7	IRGC 127421	Hsin-T'ao Yuan Ching Yu	Ind1	Taiwan
8	IRGC 125873	Psbrc50	Ind1	Philippines
9	IRGC 125951	B 6136-3-TB-0-1-5	Ind1	Indonesia
10	IRGC 125952	B_6136_E-3-TB-0-1-5	Ind1	Indonesia
11	IRGC 127435	IR 19661-364-1-2-3	Ind1	Philippines
12	IRGC 127116	Ai Jiao Zi	Indx	China
13	IRGC 125613	Jin Jun Dao	Indx	China
14	IRGC 128097	Lobang(white)	Indx	Philippines
15	IRGC 127206	Balibud	Indx	Philippines
16	IRGC 125658	Asu	Indx	Bhutan
17	IRGC 125986	IR 80310-12-B-1-3-B	Indx	Philippines
18	IRGC 127875	Uprh166	Aus	India
19	IRGC 128382	Maranhao Branco	Tropjap	Brazil
20	IRGC 127550	Landeo	Tropjap	Indonesia
21	NPGRC 00375673	Tainan 11	Tempjap	Taiwan
22	NPGRC 10A00375	Taitung 30	Tempjap	Taiwan
23	NPGRC 2011A00120	Nipponbare	Tempjap	Taiwan
24	NPGRC 00386238	Habataki	Ind	Taiwan
25	NPGRC 93A03000	Taichung Sen 10	Ind	Taiwan
26	NPGRC 93A03012	Taichung Sen 17	Ind	Taiwan
27	#	Taichung Sen Waxy 2	Ind	Taiwan

[1] The IRGC number is the accession number of the International Rice Germplasm Collection, IRRI, and the NPGRC number is the accession number of the National Plant Genetic Source Center, Taiwan.

[2] Subpopulation designation is based on Alexandrov et al. (2015)[33]. Ind1, ind2 and ind3 are three groups of *indica* rice, indx corresponds to other *indica* varieties, temp is temperate *japonica*, trop is

tropical *japonica*, temp/trop and trop/temp are admixed temperate and tropical *japonica* varieties, japx is other *japonica* varieties, Aus is *aus*, inax is admixed *aus* and *indica*, Aromatics is aromatic and admixture is all other unassigned varieties.

Table S2. The frequency (shown as a percentage) of each *OsNRAMP1*, *OsNRAMP5*, and *OsLCD* haplotype in the rice 3K panel.

Subpopulation [1]	*OsNRAMP1*		*OsNRAMP5*				*OsLCD*			Proportion
	Hap1	Hap2	Hap1	Hap2	Hap3	Hap4	Hap1	Hap2	Hap3	
Ind3	99.16%	0.42%	37.26%	14.11%	0.21%	48.42%	43.37%	10.11%	46.53%	3.41%
Ind2	100.00%	---------	44.56%	12.63%	0.70%	42.11%	70.53%	1.05%	28.07%	3.70%
Ind1	98.07%	1.45%	66.43%	3.38%	0.24%	29.95%	63.29%	18.12%	17.63%	9.53%
Indx	90.08%	9.76%	55.28%	7.48%	0.81%	36.10%	56.91%	13.82%	29.11%	2.75%
Aus	38.50%	61.00%	60.50%	1.50%	37.00%	1.00%	98.50%	---------	1.50%	12.31%
Aromatics	26.32%	72.37%	100.00%	---------	---------	---------	98.68%	---------	1.32%	2.51%
Tropjap	14.25%	85.75%	87.37%	---------	12.37%	0.27%	70.97%	13.98%	2.96%	6.62%
Jap	2.41%	97.59%	93.98%	1.20%	4.82%	---------	95.18%	---------	1.20%	20.34%
Tempjap	1.39%	97.92%	97.57%	---------	2.08%	0.35%	99.65%	---------	---------	13.70%
Subtrop	---------	99.11%	91.96%	---------	8.04%	---------	99.11%	---------	---------	9.43%
Admixture	45.63%	53.40%	76.70%	0.97%	7.77%	14.56%	85.44%	6.80%	6.80%	15.71%
Subtotal	63.46%	36.18%	65.58%	5.56%	5.16%	23.64%	70.14%	8.93%	19.05%	100.00%
Taiwan-Ind	94.44%	5.56%	77.78%	5.56%	0.00%	16.67%	38.89%	44.44%	16.67%	74.65%
Taiwan-Jap	18.87%	81.13%	77.36%	0.00%	3.77%	18.87%	94.34%	3.77%	1.89%	25.35%
Subtotal	75.28%	24.72%	77.67%	4.15%	0.96%	17.23%	52.95%	34.13%	12.92%	100.00%

[1] Ind1, ind2 and ind3 are three groups of *indica* rice, indx corresponds to other *indica* varieties, temp is temperate *japonica*, trop is tropical *japonica*, temp/trop and trop/temp are admixed temperate and tropical *japonica* varieties, japx is other *japonica* varieties, Aus is *aus*, inax is admixed *aus* and *indica*, Aromatics is aromatic and admixture is all other unassigned varieties. Proportion was calculated as the percentage that the accessions in a subpopulation accounted for in 3K-RGP.

Table S3. The functional markers for each *OsNRAMP1*, *OsNRAMP5*, and *OsLCD* haplotype.

Gene	Primer type	Primer name	Tm	Sequence	Product size
OsNRAMP1	Deletion	*OsNramp1*-F	65℃	5'-TTGGTGGAGAAGGACAGAGAA-3'	652 bp / 246 bp
		OsNramp1-R		5'-AACATTATTTGGGAGGGTGG-3'	
OsNRAMP5	SNP	*OsNramp5*-Hap2-F	60℃	5'-TCTGGACCGCACAAA-3'	HpyCH4III;
		OsNramp5-Hap2-R		5'-TCATCGGCATCAACAT-3'	460 bp / 264 bp + 196 bp
	Deletion	*OsNramp5*-Hap3-F	55℃	5'-ATGACAAGAGCTCCGAGGAG-3'	116 bp / 113 bp
		OsNramp5-Hap3-R		5'-GAGCTGAGCATCGTGAAGC-3'	
	Deletion	*OsNramp5*-Hap4-F	55℃	5'-ACCGGAATATGGAACA-3'	137 bp / 134 bp
		OsNramp5-Hap4-R		5'-CGATCAACAGATAGAAAGG-3'	
OsLCD	Deletion	*OsLCD*-Hap2-F	55℃	5'-CTTAATTAGGTGCCTACTTAGG-3'	128 bp / 122 bp
		OsLCD-Hap2-R		5'-GGTTGTTGTGCTGACTCT-3'	
	Deletion	*OsLCD*-Hap3		TaqMan Real-Time PCR	

Table S4. The frequencies of 14 Cd-mobile types in the 3K-RGP panel.

Subpopulation[1]	Type 1	Type 2	Type 3	Type 4	Type 5	Type 6	Type 7	Type 8	Type 9	Type 10	Type 11	Type 12	Type 13	Type 14	Other	Proportion[2]
Ind3	16.63%	2.53%	17.47%	3.37%	20.63%	4.00%	23.79%	--------	0.21%	0.21%	-------	-------	-------	-------	11.16%	3.41%
Ind2	31.58%	1.05%	11.93%	-------	30.88%	-------	11.23%	--------	-------	-------	-------	-------	-------	-------	13.33%	3.70%
Ind1	35.51%	14.25%	14.01%	0.48%	24.64%	3.14%	2.17%	1.21%	0.24%	-------	-------	-------	-------	-------	4.35%	9.53%
Indx	26.99%	6.67%	12.68%	0.98%	20.33%	3.58%	12.03%	4.39%	2.28%	2.11%	0.49%	0.16%	-------	0.16%	7.15%	2.75%
Aus	33.50%	--------	1.50%	-------	1.00%	-------	-------	25.00%	-------	-------	36.00%	-------	-------	-------	3.00%	12.31%
Aromatics	26.32%	--------	--------	-------	--------	-------	-------	71.05%	-------	1.32%	-------	-------	-------	-------	1.32%	2.51%
Tropjap	2.96%	7.53%	1.08%	-------	--------	0.27%	-------	57.26%	5.91%	1.34%	10.75%	-------	0.27%	0.54%	12.10%	6.62%
Jap	1.20%	--------	--------	-------	--------	-------	-------	89.16%	-------	-------	4.82%	-------	-------	-------	4.82%	20.34%
Tempjap	0.69%	--------	--------	-------	0.35%	-------	-------	95.83%	-------	-------	1.74%	-------	-------	-------	1.39%	13.70%
Subtrop	--------	--------	--------	-------	--------	-------	-------	91.07%	-------	-------	7.14%	-------	-------	-------	1.79%	9.43%
Admixture	23.30%	3.88%	1.94%	-------	11.65%	0.97%	1.94%	41.75%	1.94%	1.94%	6.80%	-------	-------	-------	3.88%	15.71%
Total	20.08%	4.86%	8.67%	0.79%	14.16%	1.85%	7.61%	27.92%	1.32%	0.73%	4.60%	0.03%	0.03%	0.10%	7.24%	100.00%

[1] Ind1, ind2 and ind3 are three groups of *indica* rice, indx corresponds to other *indica* varieties, temp is temperate *japonica*, trop is tropical *japonica*, temp/trop and trop/temp are admixed temperate and tropical *japonica* varieties, japx is other *japonica* varieties, Aus is *aus*, inax is admixed *aus* and *indica*, Aromatics is aromatic and admixture is all other unassigned varieties.

[2] Proportion was calculated as the percentage that the accessions in subpopulation accounted for in 3K-RGP.

Table S5. Analysis of variance (ANOVA) of five traits across 27 varieties and two soil Cd concentrations.

		Mean square				
**Source**	**DF**	**Root number**[2]	**Maximum Root Length**	**Shoot Length**	**Cd in Root**	**Cd in Shoot**
Replicate	2	12.01	0.39	14.0	2709 *	20 **
Variety	26	291.07 ***	47.19 ***	368.1 ***	3906 ***	46 ***
OsNramp1	(1)	58.94 *	14.59 **	809.3 ***	153	71 ***
OsNramp5	(3)	618.30 ***	4.88 *	539.1 ***	8016 ***	116 ***
OsLCD	(2)	231.98 ***	79.88 ***	612.1 ***	1087	91 ***
Haplotype	(13)	363.70 ***	37.40 ***	374.1 ***	5210 ***	73 ***
Cd condition	1	0.22	81.21 ***	365.7 ***	2132186 ***	26724 ***
Variety x Cd	26	21.09	3.08*	9.4 *	3908 ***	45 ***
Residuals	106	13.49	1.66	5.7	749	4
PVE[1]		62.5%	39.6%	50.8%	66.7%	79.3%

[1] Percentage explained variance by haplotypes of three Cd genes within the sum square of variety

[2] *, **, *** Singificant at 5%, 1% and 0.1% levels, respectively.
